# Progesterone and preterm birth

**DOI:** 10.1002/ijgo.13187

**Published:** 2020-06-10

**Authors:** Jane E. Norman

**Affiliations:** ^1^ Faculty of Health Sciences University of Bristol Bristol UK

**Keywords:** 17‐Hydroxyprogesterone acetate, Preterm birth, Preterm labor, Prevention, Progesterone

## Abstract

Progestogens (vaginal progesterone and intramuscular 17‐hydroxyprogesterone acetate) are widely recommended for women at high risk of preterm birth. Typical regimens include 17‐hydroxyprogesterone caproate (250 mg intramuscularly weekly), starting at 16–20 gestational weeks until 36 weeks or delivery for women with a singleton gestation and a history of spontaneous preterm birth, or vaginal progesterone (90‐mg vaginal gel or 200‐mg micronized vaginal soft capsules) for women with a short cervix (typically ≤25 mm). Although some randomized trials support this approach, neither of the largest trials (PROLONG for 17‐hydroxyprogesterone acetate or OPPTIMUM for vaginal progesterone) demonstrated efficacy. There are almost no data on long‐term effects, and none that shows benefit beyond the neonatal period. Although some analyses suggest the cost‐effectiveness of the approach, a cervical length screening program followed by progesterone for those with a short cervix will reduce preterm birth rates by less than 0.5%. The present review assesses evidence on the efficacy, likely impact, and long‐term effects of implementing the recommendations for progestogens in full. Clinicians and pregnant women can look forward to resolution of the conflicting views on efficacy once the Patient‐Centered Outcomes Research Initiative (PCORI)‐funded individual patient data meta‐analysis is published.

## Abstract 2

The efficacy, probable impact, and long‐term effects of progesterone or 17‐hydroxyprogesterone acetate on the prevention of preterm labor are uncertain.

## 1 BACKGROUND

**Figure 1:**
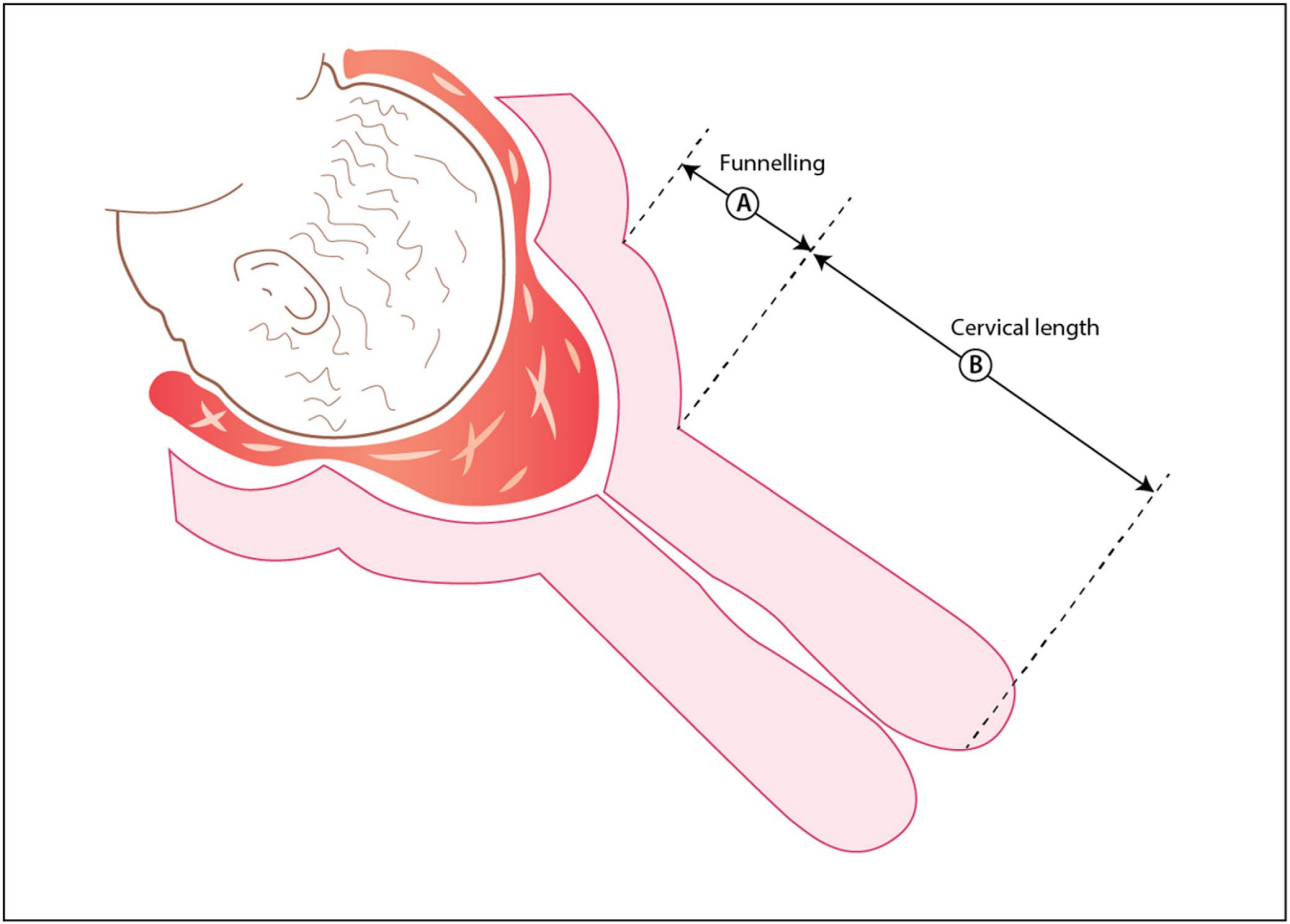
Cervical length measurement by ultrasound examination. The distance from A to B is the interval that should be reported for cervical length. The length of the “funneled” cervix should not be included.

The importance of progesterone in the maintenance of pregnancy is well established and applies across many species. Indeed, the term “progesterone” is derived from the phrase “progestational steroidal ketone.” In 2015, the International Federation of Gynecology and Obstetrics (FIGO) working group on the best practice in maternal fetal medicine published a guideline endorsing the use of progesterone to prevent preterm birth for selected women.^1^ The guideline states:

- “Sonographic cervical length measurement should be performed in all pregnant patients at 19–23^6^ weeks of gestation using transvaginal ultrasound. This can be done at the same time as the ultrasound performed for the anatomical survey (Fig. 1).”
- “Women with a sonographic short cervix (≤25 mm) diagnosed in the mid‐trimester should be offered daily vaginal micronized progesterone treatment for the prevention of preterm birth and neonatal morbidity.”
- “The progesterone formulation to be used is vaginal micronized progesterone (200‐mg vaginal soft capsules) nightly or vaginal micronized progesterone gel (90 mg) each morning.”
- “Universal cervical length screening and vaginal progesterone treatment (90‐mg vaginal gel or 200‐mg micronized vaginal soft capsules) is a cost‐effective model for the prevention of preterm birth.”
- “In cases in which a transvaginal ultrasound is not available, other devices may be used as a screening tool to measure objectively and reliably the cervical length.”

The aim of the present review is to discuss the rationale for these recommendations, their likely impact, and whether they should be updated in the light of new evidence.

## 2 ROLE OF PROGESTERONE IN PREGNANCY MAINTENANCE

Progesterone clearly plays a role in the maintenance of pregnancy. Circulating levels of progesterone rise during pregnancy: the major source (in humans) is the corpus luteum until approximately week 8 of pregnancy, and the placenta thereafter. One of the major mechanisms of progesterone action in maintaining pregnancy is inhibition of the contractions of the myometrium: research has demonstrated the relaxant effect of progesterone on myometrial strips in vitro.^2^

The importance of progesterone in maintaining human pregnancy in vivo has been demonstrated by studies administering receptor antagonists such as mifepristone (RU486). If administered in early pregnancy, mifepristone increases uterine contractility in vivo,^3^ sensitizes the uterus to the pro‐contractile effects of prostaglandins, and acts as an effective abortifacient (although it is much more effective when combined with prostaglandin).^4^ In late pregnancy, mifepristone can be used to induce labor, although its safety in the absence of intrauterine fetal death is unclear.^5^ Lastly, the withdrawal of progesterone is probably involved in the spontaneous initiation of labor at term. In many animal species, progesterone withdrawal is caused by a decrease in circulating levels of progesterone. In humans, progesterone levels are maintained until the end of pregnancy and in labor, but complex alterations in progesterone receptor activity result in a decline in progesterone receptor signaling at the time of labor onset (reviewed by Menon et al.^6^).

So, what is the evidence on which the FIGO recommendations for progesterone prophylaxis in women with a short cervix are based? To address this question, it is necessary to consider the efficacy of progesterone, the impact of such a strategy on a population basis, and the long‐term benefits and harmful effects of progesterone treatment in pregnancy.

## 3 EFFICACY OF PROGESTERONE FOR PRETERM BIRTH PREVENTION

The most recent Cochrane review on progesterone for the prevention of preterm birth (published in 2013) examined evidence on the use of any progestogen for women at risk of preterm birth either because of a previous preterm birth or because of a short cervix.^7^ Although the two risk categories were examined separately, all progestogens were considered together. For women with a previous preterm birth, the review suggested that progestogens reduce the risk of preterm birth before 34 gestational weeks [relative risk (RR), 0.31; 95% confidence interval (CI), 0.14–0.69], reduce perinatal mortality (RR, 0.50; 95% CI, 0.33–0.75), reduce the incidence of low birthweight (<2500 g; RR, 0.58; 95% CI, 0.42–0.79), and reduce neonatal death (RR, 0.45; 95% CI, 0.27–0.76). The sample size for the data on perinatal or neonatal death was 1453, whereas that for preterm birth before 34 weeks was 602.

For women with cervical shortening the review found that, among the above outcomes, only preterm birth before 34 weeks was reduced by progestogen (RR, 0.64; 95% CI, 0.45–0.90).^7^ Although there was a trend toward a reduction in perinatal mortality, a reduction in birthweight <2500 g, and a reduction in neonatal death, none of these effects was statistically significant [RR (95% CI): 0.74 (0.42–1.29), 0.92 (0.78–1.09), and 0.55 (0.26–1.13), respectively].

Further analyses in the review found no differential effects in terms of route of administration, time of commencement of therapy, or dose of progesterone for the majority of outcomes examined.^7^ Note that a comparison of different routes of administration acts as a surrogate for different types of progestogen because natural progesterone is provided in formulations given vaginally, and the synthetic formulation 17‐hydroxyprogesterone caproate is given intramuscularly.

Importantly for clinicians and for pregnant women, the regulatory status of the different progestogen formulations given for preterm birth differs. Intramuscular 17‐hydroxyprogesterone caproate (Makena) is licensed by the US Food and Drug Administration (FDA), but not the European Medicines Agency (EMA), to reduce the risk of preterm birth for women with a singleton pregnancy who have a history of singleton spontaneous preterm birth. The product license refers to a dose of 250 mg, administered intramuscularly once per week, starting between 16^+0^ and 20^+6^ gestational weeks, and continued until 37 weeks or delivery (whichever is earlier). Supporting evidence for the licensing of intramuscular 17‐hydroxyprogesterone caproate were provided by the trial of Meis et al.^8^

Although the FDA provided a license, a component of approval was the requirement for a confirmatory trial.^9^ The subsequent multicenter, randomized, double‐blind, placebo‐controlled clinical trial (“PROLONG”) recruited women with a singleton pregnancy and a history of a previous singleton spontaneous preterm delivery for randomization to either 17‐hydroxyprogesterone caproate (250 mg; 1 mL; planned n=1138) or vehicle (planned n=569) given weekly from 16^+0^ to 20^+6^ gestational weeks until 37 weeks or delivery (whichever is the earliest). The two co‐primary efficacy endpoints were preterm birth before 35 gestational weeks and a composite index of neonatal morbidity and mortality.

The results of the PROLONG study were published in October 2019.^10^ The planned sample size was achieved, with 1130 women in the 17‐hydroxyprogesterone caproate group and 578 women in the placebo group. There was no significant difference in either preterm birth before 35 weeks (11.0% vs 11.5%; RR, 0.95; 95% CI, 0.71–1.26]) or neonatal morbidity index (5.6% vs 5.0%; RR, 1.12; 95% CI, 0.68–1.61) between the two groups.

Commentators have had a mixed reaction to publication of the PROLONG study. The FDA advisory committee has suggested that 17‐hydroxyprogesterone acetate should be withdrawn from the market for the prevention of preterm delivery, although this decision has not yet been finalized. The American College of Obstetricians and Gynecologists (ACOG) and the Society for Maternal and Fetal Medicine (SMFM) have both noted that women enrolled in the PROLONG trial were at lower risk for preterm birth as compared with those in the trial of Meis et al.^8^: they were less likely to be black or to smoke, and fewer had a previous preterm birth or other risk factors for preterm birth. In view of these discrepancies, ACOG continues to endorse the use of 17‐hydroxyprogesterone acetate for the prevention of preterm birth, whereas SMFM endorses use for women with a risk profile similar to those in the trial of Meis et al.^8^

In contrast to 17‐hydroxyprogesterone caproate, vaginal progesterone is not licensed for preventing preterm birth in the United States. A submission to the FDA was prepared from data generated by a large randomized trial conducted in the United States and elsewhere.^11^ In that study, 465 women with a singleton pregnancy, a short cervix (10–20 mm) at 19^+0^ to 23^+6^ gestational weeks were randomized either to vaginal progesterone (90 mg) or placebo. The primary outcome was delivery before 33 weeks, and 235 and 223 women were available for follow‐up in each group. The progesterone group showed a significant reduction in the rate of preterm birth before 33 gestational weeks (RR, 0.55; 95% CI, 0.33–0.92) and in neonatal outcome as a composite of adverse events (RR, 95% CI 0.57, 0.33–0.99). Among the individual components of neonatal outcome, only a reduction in respiratory distress syndrome was statistically significant (RR, 0.39; 95% CI, 0.17–0.92).

Having reviewed the data from that study, the FDA chose not to provide a license for vaginal progesterone for preterm birth prevention. The FDA notes accompanying their decision state: “the statistical test used by the ‘applicant’ was inappropriate because of insufficient sample size in each strata and inconsistent treatment effects within strata,” that “the first dose, maternal age, cervical length, body mass index, and race differed notably by region,” and that “after adjusting for these covariates, the effect of progesterone in reducing preterm birth was not statistically significant”.^12^

Two large studies published since the Cochrane review in 1913 did not find a positive effect of progesterone in preventing preterm birth. For women at high risk of preterm labor, the OPPTIMUM study^13^ conducted by myself and co‐workers tested whether, relative to a placebo, prophylactic vaginal natural progesterone (200 mg daily) from 22 to 34 gestational weeks would:

- improve obstetric outcome by lengthening pregnancy and thus reducing the incidence of preterm delivery (before 34 weeks);
- improve neonatal outcome by reducing a composite of death and major morbidity;
- lead to improved childhood cognitive and neurosensory outcomes at 2 years.

The double‐masked placebo‐controlled randomized OPPTIMUM trial was conducted among 1228 women at high risk of preterm birth at 65 UK National Health Service (NHS) hospitals and one Swedish hospital between February 2009 and April 2013.^13^ The study was registered (ISRCTN14568373), and the protocol was published.^14^ Eligibility for the study (high risk for preterm birth) was conferred by a positive fetal fibronectin test performed between 22^+0^ and 24^+0^ gestational weeks, combined with a history in a previous pregnancy of any of the following: preterm birth, second‐trimester loss, premature fetal membrane rupture, or a cervical procedure to treat an abnormal smear test. In addition, women with a history of a previous spontaneous preterm birth before 34^+0^ weeks and those with a cervical length of 25 mm in the index pregnancy were eligible regardless of the fetal fibronectin test result.

The odds ratio (OR), 95% CI, and *P* values for the three primary outcomes are summarized in Table 1. Vaginal progesterone was found to have no significant effect on any of the primary outcomes. Moreover, a subgroup analysis of women with a short cervix showed no significant interaction between cervical length and outcome—in other words, women with a short cervix did not respond better (or worse) to progesterone as compared with any other women in the study.

**Table 1:** Outcomes from the OPPTIMUM study.^a^

Primary outcome	Placebo	Progesterone	aOR (95% CI)	Adjusted *P* value
Fetal death or delivery <34 wk	108/597 (18.1)	96/600 (16.0)	0.86 (0.61–1.22)	0.67
Neonatal morbidity or death	60/587 (10.2)	39/589 (6.6)	0.62 (0.38–1.03)	0.07
Cognitive composite score at 2 y	97.7 ± 17.5	97.3 ± 17.9	–0.48 (–2.77 to 1.81)	0.68

Abbreviation: aOR, adjusted odds ratio; CI confidence interval.

^a^ Taken from Ref. 13. Values are given as number/sample number (percentage) or mean ± SD.

Subsequently, the PROGRESS study^15^ randomized 787 women at high risk of preterm birth because of a spontaneous previous preterm birth to vaginal progesterone (100 mg daily) or placebo. The primary outcome, incidence of respiratory distress syndrome, was similar in both groups: 10.5% (42/402) in the progesterone group and 10.6% (41/388) in the placebo group (adjusted RR, 0.98; 95% CI, 0.64–1.49; *P*=0.912).

Thus, there are clearly conflicting data about the efficacy of progesterone for preterm birth prevention.^16^, ^17^ Some meta‐analyses, focusing exclusively on women with a short cervix, have suggested that progesterone is effective in this situation,^18^ whereas others focusing on prospectively registered studies found no difference between the groups.^19^

To address this controversy, the Patient‐Centered Outcomes Research Initiative (PCORI) has commissioned a prospective, independent comprehensive, high‐quality, individual patient data meta‐analysis on the efficacy of progesterone for preterm birth prevention. Researchers from all studies evaluating the efficacy of progestogens for preterm birth prevention have been invited to submit their data. The results are expected in early 2020, and it is likely that this project will give the most authoritative estimates of the efficacy of progesterone for preventing preterm birth and its consequences for women at high risk.

## 4 LIKELY IMPACT OF PROGESTOGENS ON PRETERM BIRTH PREVENTION

Many groups have evaluated the cost‐effectiveness of routine cervical length scanning to identify women with a short cervix, combined with prescribing progesterone for preterm birth prevention.^20^, ^21^, ^22^ Most of these studies have evaluated populations in the United States, and a majority of the studies show that such a strategy is “cost effective”. The assumptions made in one of these studies^20^ are summarized in Table 2.

**Table 2:** Assumptions around cost‐effectiveness calculations for the utility of CL scanning and progesterone prophylaxis for preventing preterm birth.^a^

Assumption	Point estimate (95% CI)
Prevalence and RR	
Prevalence of CL ≤20 mm in low‐risk population, %	0.85 (0.46–3.5)
RR of delivery at <35 wk with vaginal progesterone	0.6 (0.36–0.9)
Costs	
Cost of transvaginal sonogram, $	73.47 (50.00–340.00)
Vaginal progesterone, $	216 (100–450)

Abbreviations: CI, confidence interval; CL, confidence limit; RR, risk ratio.

^a^ Taken from Ref.^20^.

The overall analysis suggested that a policy of routine cervical length scanning (estimated to cost approximately US $3.5 million annually for pregnant women without a previous preterm birth) combined with progesterone prophylaxis for 0.85% of women with a short cervix would be cost‐effective in the United States. It would require 2.19 million vaginal sonograms, and the prescription of vaginal progesterone for 11 027 women annually; however, there would be 913 fewer preterm births and 63 fewer neonatal deaths. The total cost would be US $52 million annually. Given the costs accruing from adverse outcomes due to prematurity, the “no screen” option was most expensive. Risk‐based screening was the least expensive option, but universal screening was most cost‐effective, with an incremental cost‐effectiveness ratio of US $21 144 per quality‐adjusted life year.^20^ Another study has also demonstrated the cost‐effectiveness of routine or risk‐based screening,^21^ whereas others have suggested that routine measurement is not worthwhile in populations where the risk of spontaneous preterm birth is low.^23^, ^24^

Regardless of its cost‐effectiveness, the impact of universal cervical length screening on the rate of preterm births is trivial in the context of the overall preterm birth rate. In 2014, there were 381 659 preterm births in the United States; thus, prevention of 913 births would reduce the rate of preterm birth from 9.57% to 9.55%. Performing a similar analysis in France, Rozenberg has described this as the inverse of the Pareto principle—in other words, a large amount of effort for a small gain.^25^

There are other challenges in providing universal cervical length screening. The sonogram “cost” of US $73.47 described above probably does not include training, quality‐control programs, or equipment. In addition, without extensive training of additional providers, there will be insufficient availability of cervical length scanning to perform this analysis on a population basis. Furthermore, reproducibility is limited even when experienced observers perform measurements under standardized conditions.^26^ Others have suggested that universal treatment of all pregnant women with progestogen is the most cost‐effective strategy,^27^ although this assumes that the reduction in RR of preterm birth is the same across all risk factors and all countries: formal testing in well‐designed clinical trials is required before we can be confident that this is the case.

## 5 LONG‐TERM RISKS AND HARMS OF PROGESTERONE PROPHYLAXIS

Clinicians and pregnant women wishing to use progesterone to prevent preterm birth need to be aware of the long‐term risks and benefits. The primary rationale for preventing preterm birth is to avert adverse consequences for the newborn. Worldwide, 15 million preterm births per annum result in around 2000 neonatal deaths, and neurodevelopmental disability in nearly 1 million survivors.^28^ Data from Scotland show a reduced requirement for additional support in school for every extra week of gestational age at birth from 24 to 41 weeks.^29^ It is often assumed, therefore, that delaying preterm birth by several weeks will improve outcome. This assumption may not be true, given the role of intrauterine infection in the initiation of many preterm births,^30^ an event that can have a deleterious effect on fetal organs, particularly the brain and lungs.^31^ Long‐term follow‐up of the neonate may be needed to determine the true risks and benefits of any intervention on neurodevelopmental and other outcomes.

A good example of this is the ORACLE II randomized trial of antibiotics to prevent preterm birth among women presenting with signs and symptoms of preterm labor, but with intact fetal membranes. Although there were no major differences in outcome in the short term (and no significant impact of antibiotics on gestational age),^32^ at 7 years of age, children who had been exposed in utero to either erythromycin or co‐amoxiclav had increased rates of cerebral palsy as compared with those given placebo.^33^ There was also evidence of a stronger adverse effect among children who were exposed to both agents.

Regarding singleton pregnancy, only two studies have examined the long‐term effect of progestogens. The follow‐up to the study of 17‐hydroxyprogesterone caproate by Meis et al.^8^ showed no difference in any of the developmental domains of children assessed at approximately 2 years with approximately 80% available for assessment.^34^ A childhood developmental assessment was one of the three primary outcomes in the OPPTIMUM study, which showed no difference in cognitive composite score between the active and the placebo groups.^13^ There was a trend toward higher moderate‐to‐severe neurodevelopmental disability in the progesterone group (47/379, 12.4%) as compared with the placebo group (35/403, 8.7%), but the difference was not significant (OR, 1.48; 95% CI, 0.98–2.33; *P*=0.087).

## 6 CONCLUSION

The National Institute of Clinical Excellence in the United Kingdom,^35^ FIGO, and the SMFM in the United States all recommend the use of progestogens for women at high risk of preterm birth. The latter advises that women between 20 and 36^6^ gestational weeks receive 17‐hydroxyprogesterone caproate (250 mg intramuscularly weekly) starting at 16–20 weeks until 36 weeks or delivery for women with a singleton gestation and a history of prior spontaneous preterm birth.^36^ The two former organizations endorse the use of vaginal progesterone for women with a short cervix.

As discussed in this review, however, the evidence on efficacy for those at risk of preterm birth, impact on preterm birth rates, and long‐term effects for the baby of implementing these recommendations remains inconclusive. Clinicians and pregnant women can look forward to some resolution of the conflicting views on efficacy once the PCORI‐funded individual patient data meta‐analysis is published. Recommendations should be updated once the full details of the PCORI individual patient data meta‐analysis is in the public domain.

## AUTHOR CONTRIBUTIONS

JN wrote and revised this article, and is responsible for the final version.

## CONFLICTS OF INTEREST

The author has received funding from government and charities for research on preterm birth, stillbirth, and obesity in pregnancy. She is on a Data and Safety Monitoring Committee for GlaxoSmithKline for a trial of an agent to treat women in preterm labor, was the Chair of the NICE guideline development committee on Preterm Labor and Birth (pub. Nov 2015), and received funding from NIHR for the OPPTIMUM study of progesterone for the prevention of preterm birth.
